# Wood smoke particles from different combustion phases induce similar pro-inflammatory effects in a co-culture of monocyte and pneumocyte cell lines

**DOI:** 10.1186/1743-8977-9-45

**Published:** 2012-11-23

**Authors:** Anette Kocbach Bølling, Annike Irene Totlandsdal, Gerd Sallsten, Artur Braun, Roger Westerholm, Christoffer Bergvall, Johan Boman, Hans Jørgen Dahlman, Maria Sehlstedt, Flemming Cassee, Thomas Sandstrom, Per E Schwarze, Jan Inge Herseth

**Affiliations:** 1 Division of Environmental Medicine, Norwegian Institute of Public Health, Oslo, Norway; 2 Department of Occupational and Environmental Medicine, Sahlgrenska University Hospital and Academy, University of Gothenburg, Gothenburg, Sweden; 3 Laboratory for High Performance Ceramics, Empa, Swiss Federal Laboratories for Materials Science and Technology, Dübendorf, Switzerland; 4 Department of Analytical Chemistry, Arrhenius Laboratory, Stockholm University, Stockholm, 106 91, Sweden; 5 Department of Chemistry, University of Gothenburg, Gothenburg, Sweden; 6 Department of Respiratory Medicine and Allergy, University of Umeå, Umeå, Sweden; 7 National Institute for Public Health and the Environment, Bilthoven, the Netherlands; 8 Faculty of Health Sciences, Oslo and Akershus University College of Applied Sciences, Oslo, Norway

**Keywords:** Particulate matter, Inflammation, Wood smoke, Combustion phase, Combustion temperature, Organic fraction

## Abstract

**Background:**

Exposure to particulate matter (PM) has been linked to several adverse cardiopulmonary effects, probably via biological mechanisms involving inflammation. The pro-inflammatory potential of PM depends on the particles’ physical and chemical characteristics, which again depend on the emitting source. Wood combustion is a major source of ambient air pollution in Northern countries during the winter season. The overall aim of this study was therefore to investigate cellular responses to wood smoke particles (WSPs) collected from different phases of the combustion cycle, and from combustion at different temperatures.

**Results:**

WSPs from different phases of the combustion cycle induced very similar effects on pro-inflammatory mediator release, cytotoxicity and cell number, whereas WSPs from medium-temperature combustion were more cytotoxic than WSPs from high-temperature incomplete combustion. Furthermore, comparisons of effects induced by native WSPs with the corresponding organic extracts and washed particles revealed that the organic fraction was the most important determinant for the WSP-induced effects. However, the responses induced by the organic fraction could generally not be linked to the content of the measured polycyclic aromatic hydrocarbons (PAHs), suggesting that also other organic compounds were involved.

**Conclusion:**

The toxicity of WSPs seems to a large extent to be determined by stove type and combustion conditions, rather than the phase of the combustion cycle. Notably, this toxicity seems to strongly depend on the organic fraction, and it is probably associated with organic components other than the commonly measured unsubstituted PAHs.

## Background

Exposure to particulate matter (PM) in ambient air has been associated with effects on the pulmonary as well as the cardiovascular system. These effects include exacerbation of asthma and allergy, chronic obstructive pulmonary disease (COPD), pulmonary fibrosis, increased risk of lung cancer, atherosclerosis and acute cardiac effects [1-4]. The biological mechanisms that may explain these associations are still not resolved, but inflammation is considered to play a key role [5].

During the winter season, wood combustion is a major source of particulate air pollution in many developed countries, and the adverse health effects associated with exposure to wood smoke do not seem to be weaker than for ambient particles from other sources [6,7]. An association was recently established between PM exposure and cardiopulmonary morbidity and mortality in a community in Temuco in Chile, where 87% of PM winter emissions were estimated to originate from residential wood combustion [8]. Moreover, a study of Belgian school adolescents reported that wood fuel use was associated with increased risks of asthma, hay fever and aeroallergen sensitisation, as well as changes in systemic lung specific biomarkers [9]. Controlled human exposure to wood smoke also induced an increase of inflammatory markers in distal airways and increases in biomarkers that may be associated with systemic inflammation and cardiovascular diseases [10-12].

The potential of PM to induce biological effects seems to depend strongly on its physical and chemical properties such as size, structure and surface area, as well as components absorbed on the particle surface including metals, organic compounds, allergens and endotoxins [13,14]. The physicochemical properties of PM generated during wood combustion vary considerably depending on the combustion conditions, the combustion appliances, the type and condition of the wood, as well as the combustion phase [15-19]. The combustion of wood logs can be divided into three combustion phases. The start-up phase of the fire is characterised by low temperature and poor combustion conditions, the steady-state phase by flaming combustion, and the burn-out phase is dominated by glowing chars. It has previously been demonstrated that organic emissions, particularly those with signatures similar to levoglucosan, were strongly enhanced during the start-up phase, whereas particles emitted during the burn-out phase contained high levels of oxygenated organic species [17]. It is still unclear to what extent the combustion conditions influence the pulmonary effects of the emitted PM. However, recent *in vitro* studies indicate that particles from different combustion conditions may induce differential pro-inflammatory response patterns [20,21]. In addition, particles from poor combustion conditions with elevated organic content seem to have greater effects on both cytotoxicity and DNA damage than particles from more complete combustion conditions [20,22].

In the alveoli of a healthy lung, resident macrophages and epithelial cells lining the pulmonary surface are primary cellular targets for deposited particles. Monocytes, the precursors for alveolar macrophages, accumulate in the lung during inflammation, and have also been suggested to assist in clearance of deposited particles and to be essential in coordination of the inflammatory response [23-26]. Inflammation is a complex process involving cellular release of a range of pro- as well as anti-inflammatory mediators. The pro-inflammatory cytokines tumour necrosis factor (TNF)-α and interleukin (IL)-6, and the chemokine IL-8 are commonly used as markers for particle-induced inflammation [27-30]. TNF-α is an early inflammatory marker that seems to be an important regulator of the production and release of IL-6 and IL-8. The release of IL-6 activates the immune system and exerts multiple effects on numerous cell types including synthesis of acute phase proteins, increased antibody production in B-cells and proliferation of T-cells [31,32]. The chemokine IL-8 is an important chemo-attractant for neutrophils, which participates in the first line of cellular defence in acute inflammation. IL-8 also attracts other leucocytes, such as basophils and macrophages, and exhibits angiogenic activity [33].

The aim of the present study was to compare the inflammatory and toxic effects of wood smoke particles (WSPs) from different phases of the combustion cycle, as well as WSPs from two different combustion temperatures. A co-culture of pulmonary epithelial cells and monocytes was used as a model system. The particle samples were analysed with respect to the content of polycyclic aromatic hydrocarbons (PAHs) and selected elements. In addition, near edge x-ray absorption fine structure (NEXAFS) spectroscopy was performed for two WSPs from different combustion temperatures to determine the major groups of organic compounds present in the organic fraction. Finally, the role of the organic fraction in the observed cellular effects was investigated by comparing the responses induced by organic extracts and washed particles to the effects of the corresponding native particles. In order to relate the inflammatory and toxic potential of the WSPs to that of particles from another relevant ambient combustion source, a traffic-derived sample was also included in the study.

## Methods

### Generation and sampling of particles

WSPs from different phases of the combustion cycle were collected in an exposure chamber (Swedish National Testing and Research Institute, Borås, Sweden) during combustion of a mixture of hardwood and softwood (50% birch, 50% fir, moisture content 14–16% and 17–19% respectively) in a small cast-iron wood stove. Wood smoke was generated during three different days (10 hours/day) with wood smoke entering the chamber from the whole burning cycle on the first day (mixed wood smoke), from the start-up phase on the second day and the burn-out phase on the third day. A high-volume cascade impactor was used for collection of WSPs for the toxicological tests in two size fractions, PM_0.1–2.5_ and PM_2.5–10_[34]. The rationale for including both size fractions was that several studies report different effects of these two fractions [35,36]. The details of the generation and collection of wood smoke in the chamber are included in Additional file 1.

Two samples were included as reference particles; Wood(high-temp) and Traffic. The Wood(high-temp) sample was collected in a laboratory from a conventional stove with single-stage combustion during high-temperature incomplete combustion (approximately 700–1000°C) of birch wood with moisture content 15-20% [37]. In comparison, the particles collected in the exposure chamber were collected during medium-temperature combustion (approximately 500–800°C). The traffic sample was collected at a highway intersection in Oslo, Norway (30.000 vehicles/day, analysis published as Oslo 2, Spring, Fine (i.e. PM_2.5_), in [38]). The sample contains contributions from cars, trucks and busses driven on diesel or gasoline. The sampling and extraction procedures for the Wood(high-temp) and Traffic samples have been described in detail elsewhere [37,38]. The toxicity of these particle samples has also been investigated previously in the present co-culture model system, as described in [39]. The motivation for inclusion of these two reference samples presently was (i) to investigate the influence of combustion temperature on the toxicity of WSPs by comparing the wood smoke particles from high- and medium-temperature incomplete combustion and (ii) to compare the toxicity of WSPs with particles from another relevant ambient combustion source, i.e. road traffic.

### Physicochemical characterisation of particles

The PM_0.1–2.5_ particle samples collected in the exposure chamber were analysed for the content of a selection of PAHs and elements (see list of analysed species in Additional file 2). Gas chromatography–mass spectrometry (GC-MS) was used for analysis of the PAHs in the medium temperature PM_0.1–2.5_ WSP samples as previously described [40], whereas a modified GC-MS method was used for PAH analysis of Wood(high-temp) and Traffic [41]. The elemental composition was analysed by Energy Dispersive X-Ray Fluorescence (EDXRF) using a Mo secondary target in a three axial geometry for optimal signal to noise ratio and low detection limits [42]. The content of elements in the reference samples, Traffic and Wood(high-temp), has previously been analysed by ICP-MS, for methodological description and original data, see [38].

To determine the molecular structure of the organic fraction of the WSPs, near-edge x-ray absorption fine structure (NEXAFS) spectra were recorded for one wood smoke sample from the exposure chamber (mixed-smoke, PM_0.1–2.5_) and the reference wood smoke sample Wood(high-temp). NEXAFS spectra from the C(1s) absorption threshold typically exhibit multiple peaks indicating the presence of various carbon functional groups [43-45]. By means of reference spectra and calculations this complex spectrum can be deconvoluted into Voigt functions corresponding to particular molecular species [46]. Since the spectra are normalised, the area under the deconvolution peaks can be used to compare the content of the different molecular species in the two samples. A more extensive methodological description of the NEXAFS analysis and the assignment of the deconvolution peaks to the different groups of molecular species are included in Additional file 3.

### Cell cultures, particle preparation and exposure of cells

A co-culture of two human cell lines, A549 pneumocytes and THP-1 monocytes, was used for the toxicological experiments. The model system allowed for contact between the two cell types, and after 1 hour incubation visual inspection by microscopy revealed that the majority of the THP-1 cells rested on the A549 cells. This co-culture has previously been described in Kocbach et al. 2008 [39]. In short, A549 cells were seeded in 35 mm 6 well plates and grown to approximately 70% confluence. Then 1.6 mill THP-1 cells were added to each well immediately before exposure, corresponding to a concentration of 0.65 mill cells/ml. At the time of exposure the approximate ratio of monocytes vs. pneumocytes was 4:1. Since a limited amount of each particle sample was available, only one particle concentration was chosen for all experiments; 40 μg/cm^2^. In our previous study [39], the same model system was used to investigate the cytokine release after exposure to 10, 20 or 40 μg/cm^2^ of Traffic and Wood smoke (high-temp), samples which were included as reference sampels in the present study. Since 10 and 20 μg/cm^2^ did not increase the cytokine release significantly for both particle samples, 40 μg/cm^2^ was chosen for the present study. Exposure times of 12 or 40 hours were used as specified in the figure legends.

Particle suspensions of 1 mg/ml were prepared in cell culture medium without FBS, but supplemented with 2% dimethyl sulfoxide (DMSO), and sonicated for 30 min in a water bath. Methanol was used to aliquot the particle samples, and the subsequent evaporation of the methanol caused adherence of the particles to the tube walls. Therefore, when preparing particle stock solutions, DMSO was added to the particles before the culture medium to increase dispersion of particles into suspension. All samples were vortexed for 30 sec immediately before cell exposure. Organic extracts and washed particles were prepared by methanol extraction. A two step extraction procedure was applied where the two fractions were separated by centrifugation as described in [39]. The washed particles, but not the organic extracts, were sonicated for 30 min in a water bath before exposure. Unexposed cells, i.e. exposed to cell culture medium with DMSO, were used as controls. The final concentration of DMSO was identical in all the wells and did not exceed 0.5%. The presence of DMSO did not influence the investigated biological endpoints. After exposure, the cell culture supernatants were collected and centrifuged twice for removal of dead cells (300 x g) and particles (8000 x g), before storage at −70°C until further analysis.

### Quantification of cytokine and chemokine release

The collected cell culture supernatants were analysed by enzyme-linked immunosorbent assay (ELISA) kits (CytoSets™, Invitrogen, CA, USA or DuoSet ELISA kits, R&D Systems Inc., MN, USA) to determine the levels of the pro-inflammatory cytokines TNF-α and IL-6 as well as the chemokine IL-8. All ELISA kits were used according to the manufacturer’s manual and the detection limits for all kits were in the range 7 to 10 pg/ml. The increase in colour intensity was quantified using a plate reader (Revelation Version 4.22, Thermo Labsystems, VA, USA).

### Detection of cytotoxicity

Cytotoxicity was estimated by measuring the release of lactate dehydrogenase (LDH) from the cytosol of damaged cells into the cell culture medium, using a colorimetric cytotoxicity kit (Roche, Switzerland). The kit was supplemented with a standard with maximum concentration of 250 mU/ml (Roche). The maximal releasable LDH was determined in a suspension of unexposed cells (controls) lysed with 1% Triton x-100 and diluted with cell culture medium to the total volume applied in the well (2,4 ml). The max levels are indicated as dotted lines in the graphs showing the detected LDH levels.

As an additional indicator of cytotoxicity, particle-induced changes in the number of viable cells were detected by trypan blue exclusion. Non-adherent monocytes were removed with the supernatant and collected, while the adherent pneumocytes were removed by trypsination. Monocytes and pneumocytes were then mixed and stained with trypan blue for 3 minutes. The numbers of living cells were counted in a Bürker chamber, and the cell numbers for unexposed and exposed cells are presented as 10^6^ cells/ml.

### Cell cycle analysis

Cell cycle analysis was performed by Hoechst 33258 staining and flow cytometry in combination with curve fitting to obtain a measure of the approximate proportion of cells in each phase of the cell cycle. The cells were not synchronised prior to exposure. The WSPs and extracts introduced an artefact to the analysis. The full methodological description, the method used to account for this artefact and the results obtained from the analysis are included in Additional file 4.

### Cytokine binding to particles

The binding of IL-6 and IL-8 to a selection of the applied particle samples was investigated using a cell free test described in Kocbach et al. 2007 [47]. No significant binding was observed for the analysed particles samples. The data from the experiments are presented in Additional file 5.

### Statistical analysis

Statistical analysis was performed with GraphPad Prism (version 4.03 for Windows, GraphPad Software, CA, USA, http://www.graphpad.com). One- or two-way analysis of variance (ANOVA) was used to analyse the data sets, as specified in the figure legends, and post-tests with Bonferroni correction were used to compare groups. As indicated in the figure legends, some data were log transformed before performing ANOVA to fulfil the assumption of equal standard deviations of all sets of replicates, whereas repeated measures ANOVA was applied in some cases to account for variations in response levels between experiments [48]. Linear regression analysis was performed to investigate the influence of particle chemistry on cytokine release, toxicity and cell number. Analyses were performed for the sum of elements, Zn, K, the sum of PAHs, Benzo(b)fluoranthene, Pyrene and Benzo(ghi)perylene. Only the most significant findings of the regression analyses are presented in the text. All *p* values < 0.05 were considered to reflect statistically significant differences.

### Ethical considerations

The research reported in this paper was not carried out on humans or animals. Commercially available cell lines were applied, therefore approval from an ethics committee was not necessary.

## Results

### Particle characteristics

The chemical characterisation of the PM_0.1–2.5_ fractions from the different combustion phases is presented in Table 1, together with the data from the previous analyses of the reference samples. The PAH levels in the PM_0.1–2.5_ particles collected during the mixed-smoke and start-up combustion sessions were similar, and approximately twice as high as the levels in the particles collected during the ‘burn-out’ session. On the other hand, Wood(start-up) had a lower content of refractory elements, suggesting a lower ash-content than in the samples from the ’mixed smoke’ and ’burn-out’ sessions. However, these differences must be considered as minor, when comparing these samples with the reference samples, Wood(high-temp) and Traffic. Compared to the mixed WSPs, Wood(high-temp) contained more than five times as much PAHs, while the level of refractory elements was 3300 times higher (Table 1). Traffic had a much lower PAH content than all the WSPs, but the content of refractory elements was relatively high; approximately half of the level detected in Wood(high-temp).

**Table 1:** **Chemical characteristics of WSPs (PM**_**0.1–2.5**_**-fraction) and reference particles (traffic and wood) used for *in vitro *experiments**

			**Wood(medium-temp) PM**_**0.1–2.5**_		
	**Traffic**	**Wood(high-temp)**	**Mixed smoke**	**Start-up**	**Burn-out**
Sum of 18 PAHs (ng/mg)	48	10008	2010	2353	914
Sum of elements (ng/mg)	17423 ^a)^	32327 ^b)^	10	4	11

^a)^ ICP-MS (Cassee et al., 2003).

^b)^ ICP-MS (Cassee et al., unpublished data).

The PAH profiles provide the relative content of the individual PAHs compared to the total PAH content in each sample, and are presented in Additional file 2, Figure a, for the WSP PM_0.1–2.5_-fractions and the reference samples. The most striking difference between the PAH profiles of the five analysed samples was that Benzo(b)fluoranthene was the dominating species in the WSPs from the different phases of the medium temperature combustion, whereas the reference samples Traffic and Wood(high-temp) contained highest relative levels of Fluoranthene and Pyrene. The PAH profiles of all five samples showed similar relative levels of Benzo(a)pyrene, Benzo(e)pyrene, Benzo(a)antrachene, Chrysene, Perylene, Benzo(a)fluorene and Benzo(e)fluorene. The absolute levels of the individual PAHs in ng/mg are presented in Additional file 2, Figure c. As for the total PAH levels, most of the individual PAH levels were considerably higher in Wood(high-temp) than in the samples from medium temperature combustion, except for the levels of Benzo(b)fluoranthene which were similar for all the four WSPs.

The elemental profiles representing the content of single elements relative to the total elemental content, are presented in Additional file 2, Figure b. K and Zn were the dominating elements in all four WSPs, but the samples from the different combustion phases differed from Wood(high-temp) in that they also contained considerable amounts of Ca and Fe. The elemental profile of the Traffic sample was dominated by Fe, K and Ca.

Overall, the differences between the three phases of the wood combustion cycle were relatively small, both with respect to the sum of the measured PAHs and refractory elements, as well as the PAH and elemental profiles. There were however more evident differences between these samples and the reference samples.

The NEXAFS spectra from Wood(high-temp) and Wood(mixed-smoke, PM_0.1–2.5_) are included in Additional file 3, with a detailed interpretation of the deconvolution of the spectra. Overall, the NEXAFS spectra suggested that Wood(high-temp) had a higher content of PAHs and graphitic carbon, whereas Wood(mixed-smoke, PM_0.1–2.5_) seemed to contain more quinones, methoxyphenols and lignin decomposition products such as levoglucosan. In addition, the spectrum from Wood(high-temp) showed a potassium peak at 298 eV, whereas the Wood(mixed-smoke, PM_0.1–2.5_) spectrum did not. This pointed towards a higher ash content in the sample from the higher combustion temperature.

### Particle-induced release of TNF-α, IL-6 and IL-8

**Figure 1:**
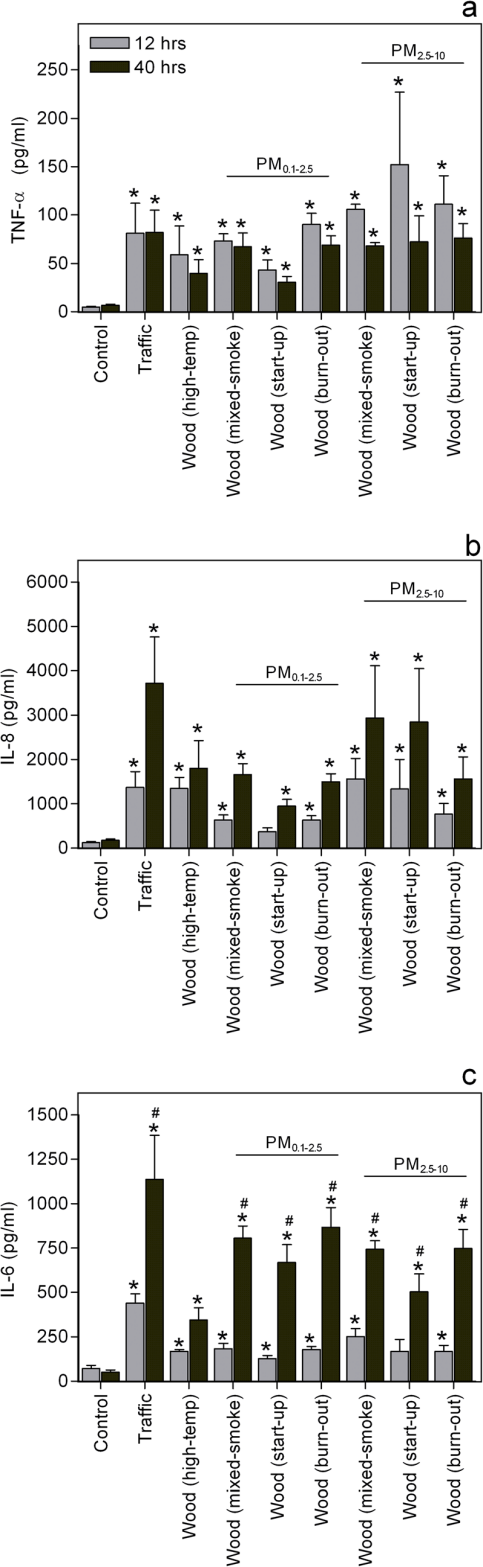
**Particle-induced release of cytokines.** Co-cultures of pneumocytes (A549) and monocytes (THP-1) were exposed to 0 (control) or to 40 μg/cm^2^ of particles for 12 or 40h, before the levels of **a**) TNF-α, **b**) IL-8 and **c**) IL-6 in cell culture supernatants were determined by ELISA. Histograms represent means ± SEM of separate experiments (n=4). * p<0.05 exposed vs. control and # p<0.05 12 vs. 40 h exposure. Statistical analyses were performed by two-way ANOVA with Bonferroni post-tests on log transformed data.

Most particle samples induced a statistically significant increase in the release of IL-6, IL-8 and TNF-α from the co-culture after both 12 hours and 40 hours exposure (*, Figure 1). With regard to exposure time, the release of IL-6 and IL-8 appeared to increase with time, whereas the release of TNF-α decreased. Significant effects of exposure time were, however, only observed for IL-6 (#, Figure 1).

The WSPs from the different phases of medium-temperature combustion did generally not differ in their potency to induce release of TNF-α, IL-6 and IL-8. The statistically significant differences between the particle samples are summarized in Additional file 6. The release of TNF-α did not differ significantly between any samples. In contrast, Traffic induced a higher release of IL-6 than most of the WSPs after 12 hours exposure, whereas Wood(high-temp) induced a lower release of IL-6 than Traffic and the medium-temperature WSPs at 40 hours. No consistent patterns were observed for IL-8.

### Particle-induced cytotoxicity

**Figure 2:**
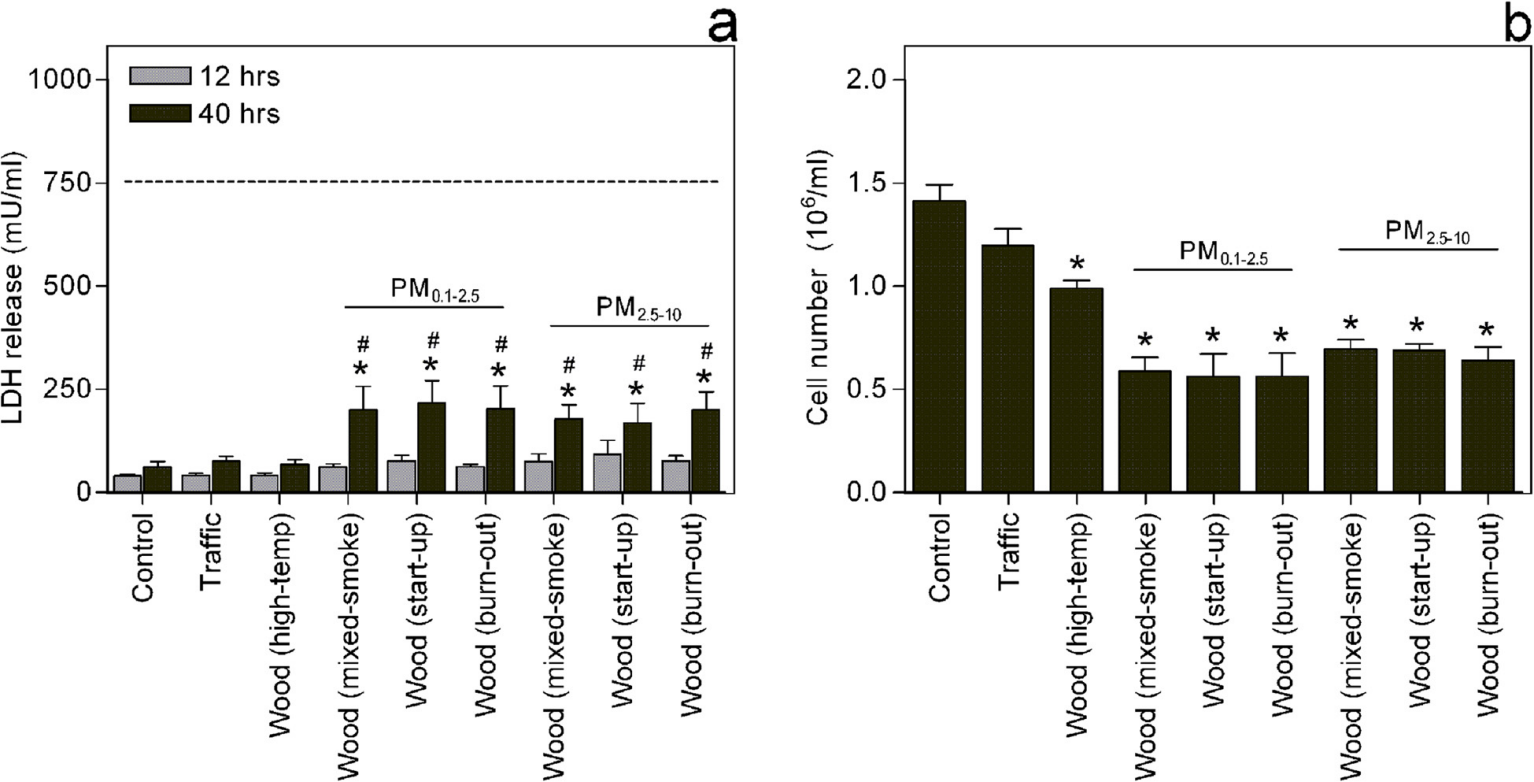
**Particle-induced changes in cytotoxicity and number of viable cells.** Co-cultures of pneumocytes (A549) and monocytes (THP-1) were exposed to particles at a concentration of 40 μg /cm^2^, before determining **a**) levels of LDH in the cell culture supernatants after 12 or 40 h exposure and **b**) the number of viable cells by tryan blue exclusion after 40 h exposure. The dotted line represents the maximum LDH level, which corresponds to the levels that would have been measured if all the cells in the wells were dead. Histograms represent means ± SEM of separate experiments (n = 4). * p<0.05 exposed vs. control and # p<0.05 12 vs. 40 h exposure. Statistical analysis was in **a**) performed by 2-way-ANOVA with Bonferroni post-tests on log transformed data and in **b**) by one-way-ANOVA with Bonferroni post-tests.

After 40 hours exposure, both fine and coarse particles from medium temperature combustion induced significant and similar increases in cellular release of LDH (*, Figure 2a). However, these LDH levels were all well below the estimated maximal release of LDH, indicating a relatively low level of particle-induced cytotoxicity. Particles from traffic and high-temperature wood combustion did not cause significant increases in the release of LDH. Consequently, the release of LDH after exposure to all the medium-temperature WSPs was significantly higher than after exposure to Traffic and Wood(high-temp) after 40 hours exposure (not indicated in the figure). The LDH release induced by the medium temperature WSPs was significantly higher after 40 hours as compared to after 12 hours exposure (#, Figure 2a).

With regard to particle-induced changes in cell numbers, exposure to all the WSPs caused significant decreases in cell numbers, in contrast to Traffic that only induced a non-significant reduction (Figure 2b). Particles collected during medium-temperature wood combustion elicited a significantly larger decrease in cell numbers than Wood(high-temp) (not indicated in figure), but there were no significant differences between the combustion phases or the particle size fractions.

### Effects of native particles versus washed particles and organic extracts

**Figure 3:**
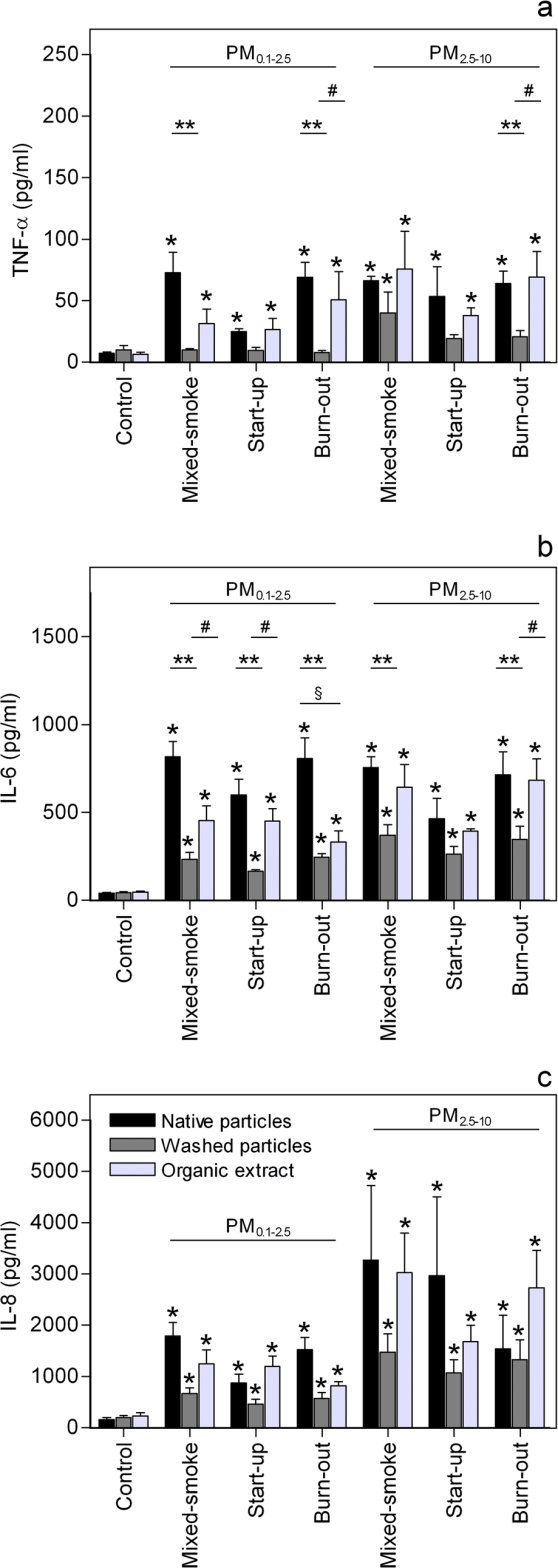
**Effect of organic extraction on the particle-induced cytokine release.** Co-cultures of pneumocytes (A549) and monocytes (THP-1) were exposed to native particles, washed particles and organic extracts at concentrations equivalent to 40 μg/cm^2^, for 40 h, before the levels of **a**) TNF-α, **b**) IL-6 and **c**) IL-8 in cell culture supernatants were determined by ELISA. Histograms represent means ± SEM of separate experiments (n = 4).* p<0.05 exposed vs. control, ** p<0.05 native vs. washed particles, # p<0.05 washed particles vs. organic extracts of particles, § p<0.05 native particles vs. organic extracts of particles. Statistical analyses were performed by two-way ANOVA with Bonferroni post-tests on log transformed data.

**Figure 4:**
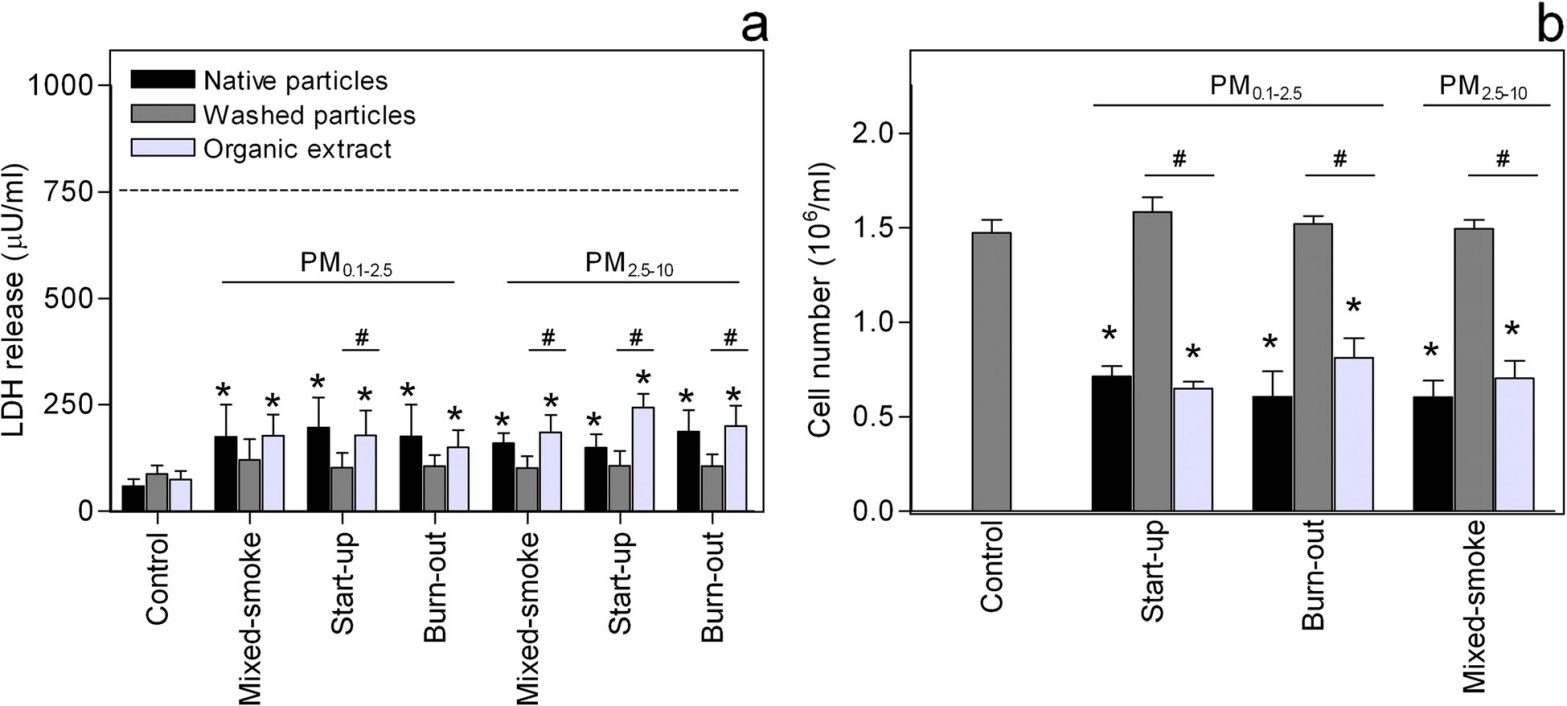
**Effect of organic extraction on changes in cytotoxicity and number of viable cells.** Co-cultures of pneumocytes (A549) and monocytes (THP-1) were exposed to native particles, washed particles and organic extracts at concentrations equivalent to 40 μg/cm^2^, for 40 h, before the levels of **a**) LDH in the cell culture supernatants and **b**) the number of viable cells were analysed. Histograms represent means ± SEM of separate experiments (n = 4). * p<0.05 exposed vs. control, # p<0.05 washed particles vs. organic extracts of particles. Statistical analysis was in **a**) performed by repeated measures 2-way-ANOVA with Bonferroni post-tests on log transformed data and in **b**) by one-way-ANOVA with Bonferroni post-tests.

The co-cultures were exposed to washed particles and organic extracts at concentrations equivalent to the concentration of native particles, to investigate the relative importance of these two fractions in the responses induced by the WSPs from the different combustion phases (Figure 3 and 4). The corresponding data for the two reference samples has been published previously in [39]. In general, native particles induced the highest cytokine release. Organic extracts of all the medium-temperature WSPs induced a significant increase in the release of TNF-α, IL-6 and IL-8, whereas the washed particles only increased the release of IL-6 and IL-8, with the exception of Wood, (mixed-smoke, PM_2.5–10_) that also increased the TNF-α release significantly. Generally, organic extracts appeared to be more potent than the corresponding washed particles, although statistically significant differences between washed particles and organic extracts were only detected for a few samples for TNF-α and IL-6 (#, Figure 3a and b). On the other hand, the TNF-α and IL-6 release induced by native and washed particles differed significantly for the majority of the samples (**, Figure 3a and b). This also supports that the organic fraction influenced the cytokine release, since particles devoid of the organic fraction induced a significantly lower response. Finally, the responses induced by native particles and organic extracts, were only significantly different for one sample (§, Figure 3b).

With respect to the release of LDH, organic extracts from all medium temperature WSPs induced a statistically significant increase, but none of the washed particles did (Figure 4a). Moreover, the organic extracts seemed to be more potent inducers of LDH release than the respective washed particles (#, Figure 4a). In line with this, the organic extracts decreased the cell numbers significantly and to a similar extent as the native particles, whereas the number of viable cells appeared unaffected by washed particles (Figure 4b).

Overall, the organic fraction seemed to account for the majority of the responses induced by native WSPs from the different combustion phases, both with respect to release of pro-inflammatory mediators and cytotoxicity. There were however no apparent differences between the combustion phases or between fine and coarse particles.

### Cell cycle analysis

Cell cycle analysis was performed by flow cytometry and curve fitting to investigate if the observed reduction in cell numbers was due to a cell cycle arrest that in turn caused a decreased proliferation. Due to particle- and extract-induced artefacts in the flow cytometry analysis, only samples of THP-1 monocytes from co-cultures exposed to organic extracts were analysed (Additional file 4, Figure 3). Exposure to organic extracts from Wood(high-temp) and Wood(mixed-smoke, PM_0.1–2.5_), but not Traffic, increased the number of cells in the S/G2 phase, suggesting an accumulation in the S/G2 phases. This is in accordance with the significant reduction in cell numbers induced by the WSPs, but not by Traffic.

### Influence of particle chemistry on cellular responses

The linear regression analysis indicated that the content of PAHs and elements had limited influence on the biological effects induced by the particles (data not shown). With some exceptions, the R^2^ values were mostly below 0.4, indicating that generally less than 40% of the variability in the responses could be explained by the content of elements and PAHs. Moreover, regression lines with slopes statistically significantly different from zero often reflected slopes that were too low to be of biological relevance. For the total elemental content the highest R^2^ value, 0.47, was determined for the cell number, with a slope of 1275 cells per 100 ng/mg increase in elemental content. A similar pattern was seen when linear regression was performed for the two most abundant elements Zn and K, with the highest R^2^ value for the cell number, 0.64 for both elements, and slopes of 5400 and 2200 cells per 100 ng/mg increase in content of Zn and K, respectively.

The linear regression analysis of the sum of the 18 measured PAHs versus the various biological endpoints generally resulted in R^2^ values below 0.1, with the exception of the release of IL-6 which gave a R^2^ value of 0.39. The slope of the corresponding linear regression line was however too low to have biological relevance. Linear regression analysis for three of the most abundant PAHs, Benzo(b)fluoranthene, Pyrene and Benzo(ghi)perylene, also gave highest R^2^ values for IL-6. Moreover, analysis of Benzo(b)fluoranthene vs, IL-8 release and cell number resulted in R^2^ values of approximately 0.25. For these single PAHs the slopes significantly different from zero could have biological relevance. For instance, IL-6 vs. Benzo(b)fluoranthene resulted in a negative slope of 70 pg/ml for 100 ng/mg PM. As a reference, the Benzo(b)fluoranthene levels varied from 5 to 810 ng/mg in the included particles samples, and the general IL-6 levels from 250 to 1250 pg/ml.

## Discussion

In order to develop more targeted abatement strategies it is important to investigate whether particle-induced health effects can be attributed to specific sources or to compounds present in the complex PM mixture. The aim of this study was to compare cellular responses to WSPs from different phases of the combustion cycle and from different combustion conditions. In addition, the effects induced by the WSPs were compared to those induced by a reference sample from traffic. WSPs from different combustion phases did not differ in their potency to induce cytokine release, cell death or reductions in cell numbers. However, particles collected during medium-temperature combustion were more toxic than the reference particles collected from traffic or during a higher wood-combustion temperature. WSPs collected during medium-temperature combustion were also more potent inducers of IL-6 than WSPs collected during high-temperature incomplete combustion.

### Differences in chemistry

Since the chemical composition of WSPs is known to depend on the combustion process and temperature [17,19,49], we expected the three WSPs from different combustion phases to differ in chemical composition. Indeed, the PAH-content of particles from the ‘mixed-smoke’ and ‘start-up’ sessions was approximately two times higher than in particles collected during the ‘burn-out’ session, but the levels of refractory elements were similar. In comparison to the differences between WSPs from medium- and high-temperature combustion, with the content of PAHs and refractory elements being five and 3300 times higher in Wood(high-temp), the differences between the medium-temperature WSPs must be considered as minor. A possible explanation for the high similarity between the WSPs from medium temperature combustion could be that the combustion conditions during collection of particles from the different phases were quite similar. The total emissions of hydrocarbons have been reported to be high in the start-up and flaming phases of the combustion cycle [17,18], but to our knowledge the variation in PAH content with the combustion cycle has not yet been fully described. Although the PAH content in the samples from the different combustion phases was relatively similar, there could be larger differences in the content of other organic compounds, such as oxy-PAHs, ketones and quinones, that was not reflected in the total PAH content. In accordance with the similar chemical composition of the medium-temperature WSPs, these samples also induced similar biological effects.

The differences in chemical composition between the high-and medium-temperature wood smoke samples reflect differences in combustion conditions. The 3300 times higher level of refractory elements in the high-temperature compared to the medium-temperature wood smoke sample is in agreement with the potassium K-shell absorption edge observed only in the Wood(high-temp) NEXAFS spectrum. This difference is also in accordance with the higher levels of refractory elements reported for increasing combustion temperatures in the literature [19,50,51]. Similarly, the five times higher PAH content in the Wood(high-temp) sample is in accordance with the NEXAFS analysis showing a higher C = C peak in Wood(high-temp) than Wood(mixed smoke). In agreement with our findings, PAH formation has been reported to increase with increasing combustion temperatures, with optimal temperatures for PAH formation between 700 and 900°C [18,51-55]. At even higher combustion temperatures, the PAHs are thermally decomposed, resulting in decreasing PAH levels during more complete combustion conditions [52,56-58]. However, other factors also influence the PAH emissions, such as oxygen supply, moisture content and residence time in the combustion zone [52,53,55], and if available these factors should be considered in the interpretation of PAH data. Although a range of studies report lower PAH levels for poor combustion conditions in line with the present findings, two recent studies report very high levels of PAHs in emissions from poor combustion conditions [20,59]. These contradicting data emphasize the importance of sufficient characterisation, not only of the emitted particulate matter applied in the toxicological studies, but also of factors determining the combustion conditions. An improved characterisation of factors determining the combustion conditions would have been feasible in the present study.

### Influence of combustion conditions

Presently, particles from incomplete combustion with medium-temperature were more potent inducers of cytotoxicity and IL-6 release than particles originating from incomplete combustion with higher temperature. In line with this, particles from poor combustion conditions have been reported to be more toxic in macrophage and fibroblast cell lines than particles from more complete combustion conditions [20,22,60]. With respect to inflammatory potential, particles from smouldering combustion were more potent inducers of macrophage inflammatory protein (MIP)-2, the murine analogue of IL-8, than particles from normal flaming combustion, whereas the release of TNF-α was similar for the two combustion conditions, and IL-6 could not be detected [20]. Similarly, intratracheal instillation of WSPs from poor combustion conditions increased the expression of MIP-2, monocyte chemotactic protein (MCP)-1 and heme oxygenase (HO)-1 mRNA in rat lung, whereas particles from more complete combustion conditions had no effect [61]. These particles from poor combustion conditions also induced the highest expression of inflammatory markers in a parallel cell culture study in two human cell lines [21]. Thus, the present finding of medium-temperature WSPs being more potent inducers of IL-6 than high-temperature particles appears to be in accordance with the previously published literature. It should however be kept in mind that the physicochemical properties vary with the combustion conditions, and based on the limited chemical analyses performed in the various toxicological studies it is difficult to compare the applied combustion conditions.

Although the present as well as previous findings suggest that both the cytotoxic and inflammatory effects of WSPs increase with decreasing combustion temperatures, it is necessary to perform more extensive toxicological studies to verify this hypothesis. Preferentially, the effects of well-characterised particles from a wider range of combustion conditions should be investigated simultaneously in the same biological model system. A recent study compared emissions from two different combustion conditions and related their *in vitro* toxicity data to the respective emission factors (mg PM_1_/MJ fuel energy) [20]. The particles had a similar inflammatory potential if compared on a mass basis, but after adjustment for the emission factors the particles from low-temperature (smouldering combustion) induced 5–20 fold higher responses compared to particles from more complete combustion conditions. This illustrates the importance of considering the exposure levels in addition to the relative toxicity on a mass basis. However, although emissions from poor combustion conditions (smouldering) are known to be much higher than emissions from more complete combustion conditions (flaming), little is known about the fate of these particles in the atmosphere and the human exposure [19]. Moreover, the relative pulmonary deposition of these two classes of particles is not known, and these gaps in knowledge preclude a full evaluation of the relative toxicity of WSPs from varying combustion conditions.

### Importance of the organic fraction

Comparison of the effects induced by native WSPs and the corresponding organic extracts and washed particles, indicated that the organic fraction was of major importance for the biological effects induced by WSPs from the different combustion phases. The washed particles did however also increase the release of IL-6 and IL-8 significantly. This could be due to an incomplete removal of the organic compounds, effects induced by the insoluble carbon core, or possibly by adsorbed metals. The organic extracts of the reference samples Traffic and Wood(high-temp) were investigated in the same series of experiments, but the data were published previously [39]. In line with the present results, the organic fraction of Wood(high-temp) accounted for the majority of the cytokine release, as well as the reduction in cell number. This may suggest that the organic fraction is of importance regardless of the combustion conditions used to generate the wood smoke. The toxicity of WSPs from varying combustion conditions has previously been linked to the condensable organic matter present in the smoke. In that study, the condensable organic matter had similar toxicity when compared on an equal mass basis, but the amount of condensable organic matter emitted was 200 times higher during poor combustion conditions as compared to complete combustion conditions [22]. The amount of reactive oxygen species (ROS) present in wood smoke emissions has also been found to increase with decreasing combustion efficiency, and the amount of ROS was linked to the content of organic carbon in the particles [62]. Although the ROS levels present in PM may not correlate with cellular effects [63], these data support that the amount of reactive chemicals in wood smoke emissions increase with decreasing combustion efficiency. Moreover, WSPs from low combustion temperature were recently found to induce higher levels of cellular ROS in monocytic and epithelial cell lines than particles from higher combustion temperatures [21].

Although the organic fraction was found to influence the release of inflammatory mediators, toxicity and cell numbers, the total PAH content was not associated with any of the biological effects in the linear regression analysis. This may point towards a limited importance of the PAHs in the inflammatory and toxic effects. Cellular studies of individual PAHs report effects on cytokine release, cytotoxicity and DNA damage [64-66]. This may suggest that these compounds contribute to the observed cellular effects after all, although the concentrations used in those studies were 10- to 1000-fold higher than the PAH concentrations during PM exposure in the present study. When linear regression was performed for the individual PAHs, these were however associated with negative slopes for IL-6 and IL-8, suggesting a decreased mediator release for increased levels of PAHs. Interestingly, PAHs have been negatively associated with release of pro-inflammatory mediators both in macrophages and mouse lung in studies using urban air particles, and the authors suggested that PAHs were associated with an immunosuppressive effect [67,68]. Overall, the R^2^ values were relatively low in the regression analysis, and it is necessary to do further experiments to investigate the role of these single PAHs in wood smoke induced effects. Generally, the present data and data from the literature [20-22] show that the *in vitro* toxicity and inflammatory potential of the particles increases with decreasing combustion temperatures, and none of these studies show a positive correlation between biological effects and PAH content. Thus, other organic compounds than PAHs seem to also be involved in the wood smoke-induced effects. Future studies of the toxicological effects of wood smoke should therefore include analysis of other organic compounds than the traditionally analysed unsubstituted PAHs like EPA PAH16.

To further characterise the organic fraction of the WSPs applied in the present study, two samples were analysed by NEXAFS. The results suggested a higher content of quinone-like compounds in the medium-temperature than the high-temperature wood smoke sample. Since the medium-temperature particles also induced the highest effects on release of IL-6 and toxicity, the content of quinone-like compounds appears to co-vary with the inflammatory and toxic effects. Interestingly, a study that applied fractionation of organic extracts of WSPs into different polarity extracts suggested that oxy-PAHs and quinones contributed to cellular oxidative stress to a larger extent that the unsubstituted PAHs [69]. Moreover, different quinones, including naphthoquinone and phenanthraquinone have been reported to induce inflammatory effects in vivo and in vitro, including recruitment of inflammatory cells to the lung and expression of pro-inflammatory mediators [70-72]. Based on the present findings and the literature, quinones may emerge as a group of organic compounds potentially involved in the biological effects of WSPs. However, further studies are necessary to confirm this hypothesis including fractionation studies for a wider range of WSPs and biological endpoints. These methods should also be combined with further chemical characterisation studies in order to search for other groups of organic compounds that might be involved in WSP toxicity.

### Reduced proliferation

The reference WSPs from high-temperature incomplete combustion have previously been associated with reduced proliferation in this co-culture [39]. Actually, the previous series of experiments showed that the cell numbers did not increase at all over time, and in combination with a lack of necrosis and apoptosis, these data suggested a complete stop in the proliferation. It could therefore be speculated that the further reduction in cell numbers induced by the medium-temperature combustion particles in the present study was due to cytotoxicity. This hypothesis is also in line with the increased release of LDH induced by all the medium-temperature WSPs, but not the high-temperature sample. Also, ambient PM and cigarette smoke have been reported to reduce cell proliferation [73,74]. In the present study, we observed an accumulation in cells in the S/G2 phase in THP-1 monocytes that could contribute to the reduced proliferation. The most severe effects on the cell cycle were observed in the A549 pneumocytes, but these histograms could not be analysed due to the severe artefact effects introduced by the organic extracts. In line with the present results, Wood(high-temp) was recently found to induce accumulation of bronchial epithelial cells in the S-phase [75], and other types of PM have been reported to induce cell cycle effects like G1 arrest or a delay in G2 or mitosis [73,75]. In a previous study, the DNA damage induced by Wood(high-temp) in mono-cultures of THP-1 and A549 was determined by the comet assay. In comparison to PM from other sources, both Wood(high-temp) and its organic extract were potent inducers of DNA damage, and WSPs also induced FPG sites, suggesting oxidative DNA damage [76]. DNA damage may cause cell cycle delays or arrests, since damaged DNA may stop the cells from passing through various checkpoints in the cell cycle [77]. Thus, oxidative DNA damage induced by organic compounds in the WSPs is a possible mechanism for the cell cycle effects observed in the present study.

### Fine vs. coarse particle size fractions

Presently, the PM_0.1–2.5_ and PM_2.5–10_ fractions induced very similar biological effects, and few statistically significant differences were detected. This is in contrast to *in vitro* studies of other particle samples in which the PM_2.5–10_ fractions seem to be more potent than the PM_2.5_ fractions in inducing a pro-inflammatory response [35,36]. Possible explanations include a higher content of microbial components like endotoxin in the coarse fraction, and also that different sources contribute to the two size fraction causing differences in physicochemical properties [78,79]. A possible explanation for the similar cellular responses induced by the fine and coarse particle fractions presently might be that these two fractions of wood smoke have similar physicochemical properties. During collection of the applied WSP samples the PM_0.1–2.5_ fraction accounted for 79–86% of the total PM_10_ emissions (data not shown), which is in agreement with the PM_1_ to PM_10_ ratio of 0.8 reported for the PM mass emissions from both smouldering and flaming combustion conditions [80]. Since emissions from residential wood combustion are dominated by the PM_2.5_ fraction both with respect to number and mass concentrations [80], characterisation of the toxicity of the PM_2.5_ fraction rather than the PM_2.5–10_ fraction appears to be feasible in future toxicological studies.

### Wood smoke vs. traffic

We have previously compared *in vitro* effects of particles collected from wood smoke and traffic and concluded that traffic-derived particles were more potent in inducing inflammatory responses, while WSPs were more potent in reducing cellular proliferation [39]. In the present study, the pro-inflammatory potential of traffic-derived particles was not consistently higher, since wood smoke and traffic particles induced a similar release of inflammatory mediators. Overall, our present and previous findings suggest that wood smoke and traffic particles have a similar pro-inflammatory potential *in vitro*, but that WSPs seem more cytotoxic and more potent in reducing proliferation than traffic derived particles. However, the pulmonary effects depend on the deposited dose, and WSPs from poor combustion conditions were recently found to deposit to a lower extent in the lungs than diesel exhaust particles, providing different pulmonary doses [56]. To compare the effects of particles from the two sources it is therefore necessary to perform inhalation studies accounting for possible differences in pulmonary deposition. In the published inhalation studies in animals and humans, the authors report that PM from diesel exhaust and wood smoke induce effects of similar magnitude [10,11,81,82], whereas a more recent study suggests that WSPs may be less potent [83]. It should however be kept in mind that the large variation in the physicochemical properties of particles emitted from each source precludes the comparison of the biological effects, since the differences reported will depend strongly on the composition of the particles included in the study.

### Limitations

The applied particle concentration of 40 μg/cm^2^ is likely to be higher than the average deposition on the lung surface during normal ambient concentrations. Particle deposition in the human airway is highly uneven, but the retention for fine particles has been suggested to be highest in the proximal alveolar region [30,84,85]. The slow particle clearance in the alveolar region, with biological half-lives up to 120 days, also contributes to increased particle exposure [86]. The applied particle concentrations might be relevant for specific regions of the lung during long time exposure to high concentrations of air pollution. The single particle concentration applied presently was chosen based on results obtained in a previous study in which the effects of lower concentrations of particles with similar physicochemical properties did not cause a significant increase in cytokine release in the same co-culture model. That study showed a monotonous increase in cytokine release with increasing particle concentrations. Thus, although inclusion of a concentration-dependent response to the present particle samples would have provided a more robust data set, inclusion of more, lower, particle concentrations would most likely have given the same overall picture of the inflammatory potential of the analysed samples. Previously, the cytokine release induced by Wood(high-temp) was found to be attenuated for increasing exposure times, therefore inclusion of two time-points was considered more important in the present study than inclusion of several different particle concentrations. For risk assessment purposes a more complete evaluation of the relative toxicity of WSPs is necessary, both due to the limitation of applying only one particle concentration, but also due to the limitations inherent in *in vitro* studies, including altered cellular characteristics of cell lines and limited communication with other cells compared to cells in tissue.

## Conclusion

WSPs from three different phases of the combustion cycle induced very similar effects on release of pro-inflammatory mediators, cytotoxicity and decrease in cell number in a co-culture of monocytes and lung epithelial cells. The particles from medium-temperature combustion were, however, more cytotoxic than the particles from high-temperature incomplete combustion and also induced a higher release of IL-6. Thus, stove type and combustion conditions may be more important than phase of combustion cycle with respect to the toxicity of the emitted particles.

The organic fraction was the most important determinant for the biological effects induced by the different wood combustion particles, but the responses induced by the organic fraction could generally not be linked to the PAH content. This suggests that other organic compounds were also involved in the biological effects, with quinone-like compounds as a possible candidate. Based on the present results and the literature we therefore recommend that future toxicological studies include analysis of other groups of organic compounds, in addition to the traditionally analysed PAHs.

Although the present study suggests that particles from medium-temperature combustion have a higher toxic and inflammatory potential than particles from more complete combustion conditions, a differential deposition of PM from varying combustion conditions might influence the deposited dose. In order to properly evaluate the relative toxicity of WSPs from varying combustion conditions it is therefore of major importance to perform inhalation studies.

## Competing interests

The authors declare that they have no competing interests.

## Authors' contributions

AKB coordinated the study. GS was responsible for generation and collection of WSPs, and MS participated in the particle collection. TS and FC coordinated the extraction and aliquotation of particles. JB performed the EDXRF analysis, while RW and CB were responsible for the PAH analysis. AB carried out the NEXAFS analysis and interpretation. AKB and JIH designed and performed the *in vitro* experiments and the analysis of biological effects. HJD performed the flow cytometry analysis. AKB interpreted the cell cycle data, performed the statistical analyses, and prepared the figures. AKB, JIH and AT had the main responsibility for preparation of the manuscript, but all authors were involved in drafting the manuscript or revising it critically. All authors read and approved the final manuscript.

## Supplementary Material

Additional file 1 Generation of wood smoke in exposure chamber. Click here for file

Additional file 2 Chemical characterisation of particle samples. Click here for file

Additional file 3 NEXAFS. Click here for file

Additional file 4 Cell cycle analysis. Click here for file

Additional file 5 Cytokine binding to particles. Click here for file

Additional file 6 Statistical comparison of cytokine release induced by the various particle samples. Click here for file
