# Supplementary material for: Wood smoke particles from different combustion phases induce similar pro-inflammatory effects in a co-culture of monocyte and pneumocyte cell lines
Source: Part Fibre Toxicol. 2012 Nov 23;9:45. doi: 10.1186/1743-8977-9-45 (PMC3544657; doi:10.1186/1743-8977-9-45)
Supplement: Additional file 1 — Generation of wood smoke in exposure chamber. [file 1743-8977-9-45-S1.doc]

## Additional file 1:

## Generation, sampling and characterisation of wood smoke particles from the exposure chamber.

## Generation of wood smoke in the chamber

Wood smoke was collected in an exposure chamber, built at the Swedish National Testing and Research Institute, in Borås, Sweden, with dimensions 44 m² x 2.9 m and walls covered by Teflon-impregnated glass-fiber fabric. A more detailed description of the exposure chamber and principles of wood smoke generation have been reported previously [1]. Wood smoke was generated in a small cast-iron wood stove placed just outside the chamber. A partial flow of the generated wood smoke was mixed with indoor air (filtered using a high-efficiency particulate air filter) to the target concentration. A mixture of hardwood and softwood was used (50 % birch, 50 % fir, moisture content 14-16 % and 17-19 % respectively). A 2.5-3 kg batch combining small and large logs was ignited, and approximately every 40 minutes another three logs of 1.5 kg were added until the session was over. Wood smoke was generated during three different days (10 hours/day) with wood smoke entering the chamber from the whole burning cycle (mixed wood smoke) on the first day, from the start-up phase on the second day and the burn-out phase on the third day. When wood smoke was collected from the start-up phase of the combustion cycle, smoke was supplied to the chamber for 12-14 minutes starting immediately after new wood logs were added, whereas smoke was supplied for 15 minutes starting 25 minutes after wood was added for the burn-out phase. The aim was to generate a PM_2.5_ mass concentration in the chamber of 250-300 µg/m^3^ in each session.

## Collection of particles for toxicological experiments

A high-volume cascade impactor was used for collection of wood smoke particles for the toxicological tests [2]. Two particle fractions, PM_0.1-2.5_ and PM_2.5-10_, were collected by impaction onto polyurethane foam (PUF). The applied sampling cut-of for the fine fraction was 0.18 µm. These PM fractions were extracted from the PUF with methanol, based on a method described by Salonen and co-workers [3], and stored at -20°C to prevent changes in the composition due to evaporation or chemical reactions.

## Characterisation of the wood smoke generated in the chamber

In order to characterize the wood smoke generated in the exposure chamber, a range of measurements were performed in parallel with the collection of particles for toxicological experiments. The same measurements were performed in a parallel human inhalation study conducted under similar combustion conditions. These analyses therefore make it possible to relate the present toxicological findings to the results from the human inhalation study. As indicated in Table 1 these measured parameters included PM_1_ and PM_2.5_ mass concentrations, particle number concentrations, size distributions of particles (0.007–6.7 µm) and the content of BC (black carbon) and a selection of particulate PAHs. The particle mass concentration was determined and then analysed for its content of BC by an optical method (Magee Inc OT21 Transmissiometer) and for its PAH content using gas chromatography and mass spectrometry (GC-MS) [4, 5].

**Table 1: Physiochemical characteristics of the wood smoke generated during the different combustion sessions in the exposure chamber.** Results represent time-weighted averages of the online measurements (TEOM, ELPI), or means of replicate filters (PM_1_ mass, PAHs).

|  | Mixed smoke | Start-up | Burn-out |
| --- | --- | --- | --- |
| PM_2.5_ (µg/m^3^) * | 205 | 237 | 277 |
| PM_1_ (µg/m^3^) ^#^ | 223 | 267 | 342 |
| PM_2.5_ (#/cm^3^)* | 83960 | 51120 | 120650 |
| Ultra fine particles (%)* | 50 | 39 | 37 |
| BC (µg/m^3^) ^¤^ | 96 | 96 | 185 |
| Sum of PAHs (ng/m^3^) ^¤^ | 135 | 188 | 84 |

## * time-weighted average (TEOM)

## ^#^ mean of 6 measurements for each phase

## ^¤^ mean of 2 measurements for each phase

The sum of PAHs represents the measurements of eight individual PAHs; Chrysene, Benzo(b)fluoranthene, Benzo(k) fluoranthene, Benzo(a)pyrene, Ideno(1,2,3-c,d)pyrene, Benzo(g,h,i)perylene, Dibenz(a,h)anthracene) and Benzo(g,h,i)perylene.

The particles generated during the ’mixed smoke’ and ’start-up’ sessions had similar BC content, but the ’mixed smoke’ had a higher percentage of ultrafines and a somewhat lower PAH content than the ‘start-up’ smoke. Compared to the particles generated during the ’mixed smoke’ and ’start-up’ sessions, the particles from the ’burn-out’ session had a higher BC content, but a considerably lower PAH content. This is in agreement with the PAH data for the samples collected for toxicological experiments, where mixed smoke and start up had a considerably higher PAH content (Table 1, main paper).

One paper from the human inhalation study concerning effects on the airways of human volunteers has been published [6]. Exposure to wood smoke from the start-up, but not the burn-out phase, increased the concentration of Clara cell protein 16 (CC16) in serum after 4 hours, and in urine the next morning. Comparison of the measurements done in the chamber to characterise the generated smoke in the two series of experiments (human inhalation study; collection of PM for *in vitro* toxicity studies) reveal that the properties of the particles seem to be fairly similar when judged as total PAH or BC per particle mass (PM_2,5_). However, the amount of PM_2,5_ generated during the inhalation study was considerably higher during the start-up session (295 µg/m^3^) as compared to the burn-out session (146 µg/m^3^), whereas the particle concentrations applied in the *in vitro* study was identical for the two phases. This precludes the comparison of the data from the two series of experiments.

Reference List

1. Sällsten G, Gustafson P, Johansson L, Johannesson S, Molnar P, Strandberg B, Tullin C, Barregard L: **Experimental wood smoke exposure in humans.** *Inhal Toxicol* 2006, **18:**855-864.

2. Steerenberg PA, van Amelsvoort L, Lovik M, Hetland RB, Alberg T, Halatek T, Bloemen HJ, Rydzynski K, Swaen G, Schwarze P, Dybing E, Cassee FR: **Relation between sources of particulate air pollution and biological effect parameters in samples from four European cities: an exploratory study.** *Inhal Toxicol* 2006, **18:**333-346.

3. Salonen RO, Pennanen A, Halinen AI, Hirvonen MR, Sillanpaa M, Hillamo R, Karlsson V, Kosentalo T, Aarnio P, Ferguson S, Koutrakis P: **A chemical and toxicological comparison of urban air PM10 collected during winter and spring in Finland.** *Inhal Toxicol* 2000, **12:**Suppl. 103.

4. Kliucininkas L, Martuzevicius D, Krugly E, Prasauskas T, Kauneliene V, Molnar P, Strandberg B: **Indoor and outdoor concentrations of fine particles, particle-bound PAHs and volatile organic compounds in Kaunas, Lithuania.** *J Environ Monit* 2011, **13:**182-191.

5. Johannesson, S., Bergemalm-Rynell, K., Strandberg, B., and Sallsten, G.: **Indoor concentration of fine particles and particle-bound PAHs in Gothenburg, Sweeden.** In INHALED PARTICLES X, Book series: journal of Physics Conference Series; 2009.

6. Stockfelt L, Sallsten G, Olin AC, Almerud P, Samuelsson S, Johannesson S, Molnar P, Strandberg B, Almstrand AC, Bergemalm-Rynell K, Barregard L: **Effects on airways of short-term exposure to two kinds of wood smoke in a chamber study of healthy humans.** *Inhal Toxicol* 2012, **24**:47-59.
