# Supplementary material for: Wood smoke particles from different combustion phases induce similar pro-inflammatory effects in a co-culture of monocyte and pneumocyte cell lines
Source: Part Fibre Toxicol. 2012 Nov 23;9:45. doi: 10.1186/1743-8977-9-45 (PMC3544657; doi:10.1186/1743-8977-9-45)
Supplement: Additional file 2 — Chemical characterisation of particle samples. [file 1743-8977-9-45-S2.doc]

**Additional file 2:**

**Chemical characterisation of particles samples**

**Results presented as relative content of PAHs and elements (i.e. in %).**

a

b

#### Figure a-b: Relative content of selected PAHs and elements of WSPs (PM_0.1-0.1-2.5_-fraction) and reference particles (traffic and wood) used in *in vitro* experiments. a) The relative content of a selection of 18 PAHs for the PM_0.1-2.5_ samples collected during the different combustion phases and the two reference samples. The relative content was calculated by dividing the content of each compound by the sum of the 18 PAHs for each sample (Table 1, main paper) and subsequent multiplication by 100. b) The relative content of the 6 analysed elements for the PM_0.1-0.1-2.5_ samples collected during the different combustion phases and the two reference samples. The relative content was calculated by dividing the content of each element on the sum of the 6 elements for each sample (Table 1, main paper) and subsequent multiplication by 100.

**Results presented as absolute values of PAHs and elements (i.e. as ng/mg).**

c

d

**Figure c-d: Data from chemical characterisation presented as ng/mg.** a) The levels of the analysed PAHs in the samples presented as ng/mg. The levels of PAHs in traffic are too low to be visible in the figure, but the figure illustrates that the Benzo(b)fluoranthene levels are similar in the four wood smoke samples. b) The levels of the analysed elements in the samples in ng/mg. The elemental content in the medium temperature wood smoke samples is too low to be visible in the figure, but the figure illustrates that Wood(high-temp.) is characterised by K and Zn, whereas Fe is the dominating element in Traffic.
