# Supplementary material for: Wood smoke particles from different combustion phases induce similar pro-inflammatory effects in a co-culture of monocyte and pneumocyte cell lines
Source: Part Fibre Toxicol. 2012 Nov 23;9:45. doi: 10.1186/1743-8977-9-45 (PMC3544657; doi:10.1186/1743-8977-9-45)
Supplement: Additional file 3 — NEXAFS. [file 1743-8977-9-45-S3.doc]

# Additional file 3:

# NEXAFS spectroscopy: Results, discussion and full methodological description

**Results**

#### Figure 1: C(1s) NEXAFS spectra of Wood(high-temp) and Wood(mixed-smoke, PM_0.1-2.5_). The spectra are normalised to unity at the high energy tail and deconvoluted into Voigt functions for particular molecular motifs. The peak assignment is based on [1, 2] as described in methodological description below. The resonances accounted for in the deconvolution of the high-temperature WS spectrum were adopted in the deconvolution of the spectrum of the Wood(mixed-smoke, PM_0.1-2.5_).

Figure 1 shows the NEXAFS spectra from Wood(high-temp) and Wood(mixed-smoke, PM_0.1-2.5_), where the deconvoluted peaks of different colours correspond to particular molecular motifs. The spectra obtained from the two samples have cumulative peaks at 285 eV of similar heights, but the deconvoluted peaks suggest that different molecular species add up to these cumulative peaks. Wood(high-temp) has a higher deconvolution peak at 285 eV corresponding to the aromatic C=C which is assigned to PAHs and graphite-like carbon, while Wood(mixed-smoke, PM_0.1-2.5_) has a considerably larger peak from the C=C groups in benzoquinone at around 284.3 eV than Wood(high-temp). The area of the conjugated benzoquinone C=O peak is similar for the two samples.

The cumulated peaks at around 287 and 288 eV corresponding to C-OH and COOH are considerably higher in the Wood(mixed-smoke, PM_0.1-2.5_) spectrum than those in the Wood(high-temp) spectrum. The C-OH groups that are characteristic for lignin combustion products such as levoglucosan would peak around 287 eV [2, 3], while methoxyphenols would cause a peak around 288 eV [4]. Thus, the spectra suggest that Wood(mixed-smoke, PM_0.1-2.5_) might contain more levoglucosan and methoxyphenols than Wood(high-temp). Finally, we notice that the Wood(high-temp) spectrum shows a potassium peak at 298 eV, whereas the Wood(mixed-smoke, PM_0.1-2.5_) spectrum shows no such peak, revealing a higher ash content in the sample from higher combustion temperature.

**Discussion**

The NEXAFS spectrum obtained from medium-temperature combustion resembled previously published wood smoke spectra with a small aromatic/graphitic peak at 285 eV and a much more pronounced peak at 287 eV, likely from the C-OH group in methoxy-phenols [2, 5]. In contrast, the spectrum from Wood(high-temp) showed closer resemblance to spectra obtained from diesel exhaust particles, with a strong aromatic/graphitic peak at 285 eV and a low phenolic peak at 287 eV. NEXAFS spectroscopy has the potential to distinguish between combustion particles originating from different sources [2, 5, 6], although source attribution may not always be unambiguously possible. The present data confirm that similar spectra may be obtained from different combustion sources, since one of the WSPs provided a NEXAFS spectrum that was difficult to distinguish from diesel exhaust spectra. Application of NEXAFS in future studies with a larger array of samples would allow for a correlation or regression analysis to link the organic chemistry to the particle toxicity. The organic fraction of PM is a complex mixture comprising thousands of compounds. In the search for the organic compounds involved in the toxicity of PM it may be feasible to identify groups of compounds involved in the toxicity rather than individual compounds, and in this context NEXAFS may provide a promising tool in future toxicological studies.

**Methodological description**

Near-edge x-ray absorption fine structure (NEXAFS) spectra were recorded at beamline 9.3.2 at the Advanced Light Source in Berkeley, California, for one wood smoke sample from the exposure chamber (mixed-smoke, PM_0.1-2.5_) and the reference wood smoke Wood(high-temp) In order to prevent sample charging, the wood smoke particles were roll-pressed into a thin indium foil to provide good electric contact. The size of the indium foil pieces was about 5 x 5 mm in area after roll-pressing. The pieces were then put on double sticky carbon tape, which was readily attached on the CuBe-sample holder. The sample holder was transferred via a load lock tube into the ultra high vacuum (UHV) chamber. The residual pressure in the UHV recipient during measurement was typically 1E-5 Torr. The energy resolution of the beam line x-ray optics was set to 1/10.000, and the spectra were recorded in steps of 0.1 eV from 274 eV to 300 eV. The spectra were normalized to unity intensity at 300 eV.

In order to speciate the molecular structure of the carbon compounds in the samples NEXAFS spectroscopy was performed at the C(1s) absorption threshold. These spectra typically exhibit multiple peaks indicating the presence of various carbon functional groups. By means of reference spectra and calculations, it has become possible to determine the contribution of various molecular structures to complex mixtures of organic materials such as airborne particulate matter containing carbonaceous species [2, 6]. In this process a complex spectrum is deconvoluted into Voigt functions using WinXAS and peaks corresponding to particular resonances are assigned to particular molecular species based on reference spectra [7]. Since the spectra are normalised, the area under the deconvolution peaks can be used to compare the content of the different molecular species in the two samples.

In a previous study, C(1s) NEXAFS spectra from a range of wood smoke samples were studied [2]. The NEXAFS peak assignment performed for the two wood smoke samples in the present study is based on early work by Cody et al., and our previous NEXAFS analysis of wood smoke samples [1, 2]. The resonances accounted for in the deconvolution of Wood(high-temp) were applied in the deconvolution of the spectrum of Wood(mixed-smoke, PM_0.1-2.5_).

*Peak assignment*

Generally, the intensity peak from around 283 eV to around 286 eV originates from transitions of carbon with double bonds in an aromatic setting, such as for example graphite, quinones, phenols, and polycyclic aromatic hydrocarbons (PAH), whereas aliphatic carbon moieties such as carboxyl or hydroxyl are typically found in the energy range from 286 to 289 eV [1, 8]. Table 1 provides an overview of the different deconvolution peaks with respect to energy, functional moiety, corresponding group of compounds, molecular structure of example compound and colour code used in the graphs in Figure 1.

**Table 1:** Assignment of NEXAFS peak positions and corresponding molecular species.

| **Energy [eV]** | **Functional moiety** | **Group of compounds** | **Molecular structure of example compound** | **Colour code for deconvolution peaks** |
| --- | --- | --- | --- | --- |
| 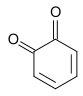284 | C=C | Quinones,  e.g. benzoquinone |  | Blue  284 |
| 285 | C=C | Graphite and aromatic e.g. pyrene | 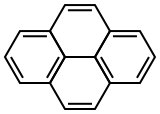 | Green 285 |
| 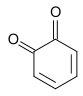286 | C=O | Conjugated phenolic compounds like quinones and ketones |  | Blue286 |
| 287 | C-OH | Phenolic , e.g. levoglucosan.  This is C-OH in an aromatic setting | 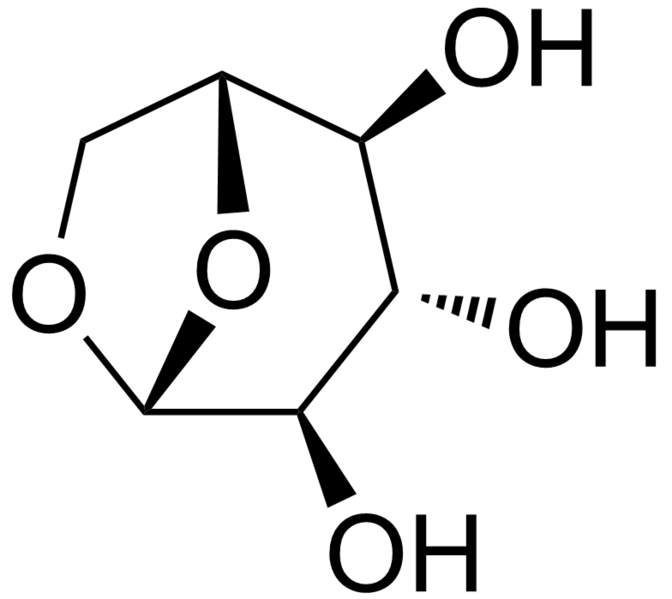 | Green 288 |
| 286 | C-OH | Aromatic C-OH  e.g. methoxyphenol | 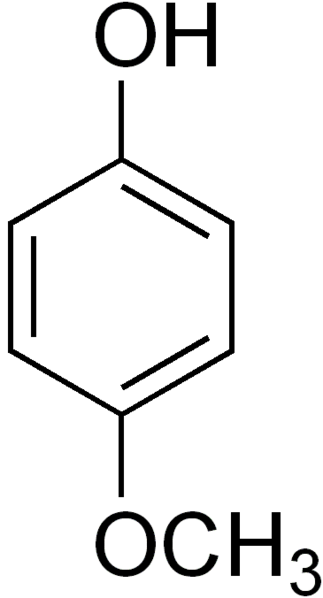 | Yellow 287 |
| 288 | COOH | Carboxyl , such as in humic and fulvic acids, e.g. formic and acetic acids | 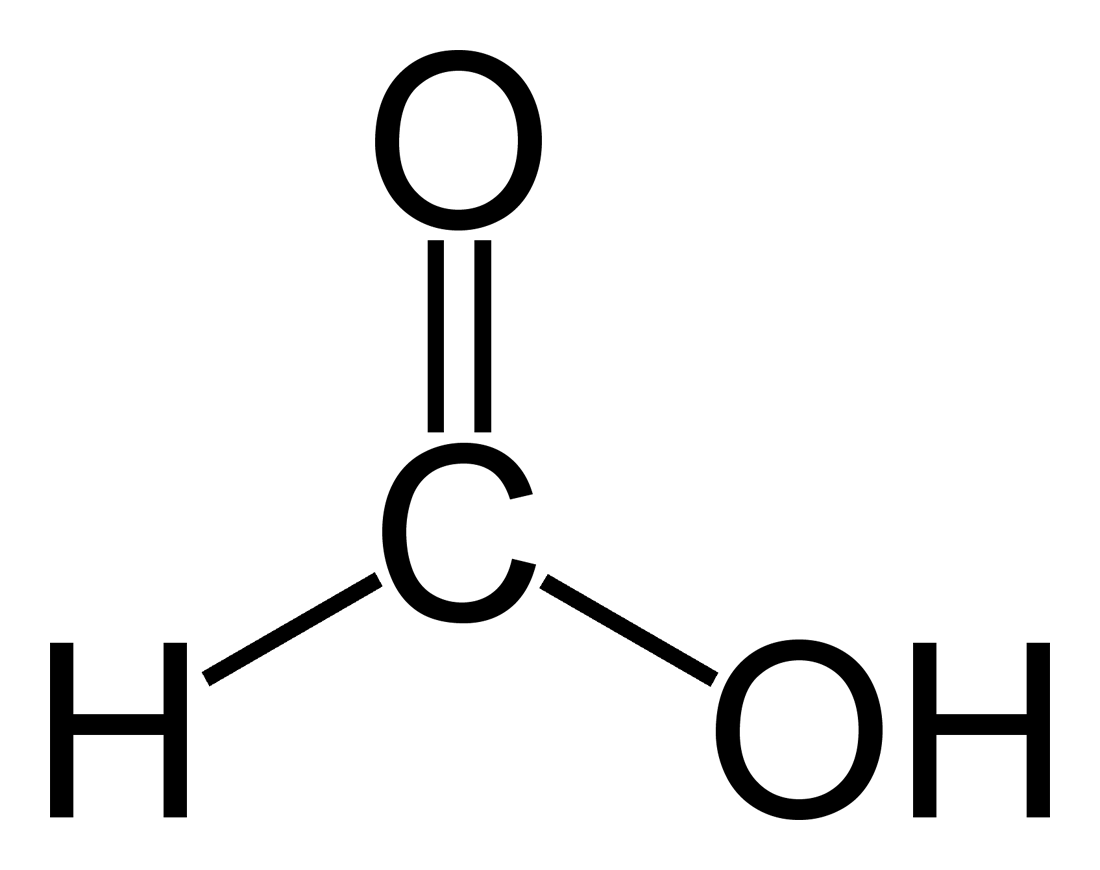 | Orange 289 |

**4. List of references**

1. Cody GD, Ade H, Wirick S, Mitchell GD, Davis A: Determination of chemical-structural changes in vitrinite accompanying luminescence alteration using C-NEXAFS analysis. *Org Geochem* 1998, 28:441-455.

2. Braun A, Huggins FE, Kubatova A, Wirick S, Maricq MM, Mun BS, McDonald JD, Kelly KE, Shah N, Huffman GP: Toward distinguishing woodsmoke and diesel exhaust in ambient particulate matter. *Environ Sci Technol* 2008, 42:374-380.

3. Jordan TB, Seen AJ: Effect of airflow setting on the organic composition of woodheater emissions. *Environ Sci Technol* 2005, 39:3601-3610.

4. Kubatova A, Lahren TJ, Beránek J, Smoliakova IP, Braun A, Huggins FE: Significance of extractable organic carbon and its differentiation by polarity in air particulate matter. *Aero Sci Technol* 2009, 43:714-729.

5. Vernooij MG, Mohr M, Tzvetkov G, Zelenay V, Huthwelker T, Kaegi R, Gehrig R, Grobety B: On source identification and alteration of single diesel and wood smoke soot particles in the atmosphere; an X-ray microspectroscopy study. *Environ Sci Technol* 2009, 43:5339-5344.

6. Takahama S, Gilardoni S, Russell LM, Kilcoyne ALD: Classification of multiple types of organic carbon composition in atmospheric particles by scanning transmission X-ray microscopy analysis. *Atmospheric Environment* 2007, 41:9435-9451.

7. Braun A: Carbon speciation in airborne particulate matter with C(1s) NEXAFS spectroscopy. *J Environ Monit* 2005, 7:1059-1065.

8. Urquhart SG, Ade H: Trends in the carbonyl core (C 1S, O 1S) --> ð*C=O transition in the N spectra of organic molecules. *J Phys Chem B* 2002, 106:8531-8538.
