# Supplementary material for: Wood smoke particles from different combustion phases induce similar pro-inflammatory effects in a co-culture of monocyte and pneumocyte cell lines
Source: Part Fibre Toxicol. 2012 Nov 23;9:45. doi: 10.1186/1743-8977-9-45 (PMC3544657; doi:10.1186/1743-8977-9-45)
Supplement: Additional file 4 — Cell cycle analysis. [file 1743-8977-9-45-S4.doc]

**Additional file 4:**

**Cell cycle analysis: results, methodological description and correction for artefacts introduced by organic extracts**

**Results**

Figure 1 shows data from cell cycle analysis for THP-1 monocytes from co-cultures exposed to organic extracts, after correction for the artefact introduced by the organic extract (as described below). The histograms recorded from the exposed A549 cells were not analysed since the histograms were more distorted and we were not confident in applying the method used to account for artefacts for data from these cells (see further explanation below).

Exposure to organic extracts from Wood(high-temp) and Wood(mixed-smoke, PM_0.1-2.5_), but not Traffic, increased the number of cells in the S/G2 phase, suggesting an accumulation in the S/G2 phases.

**
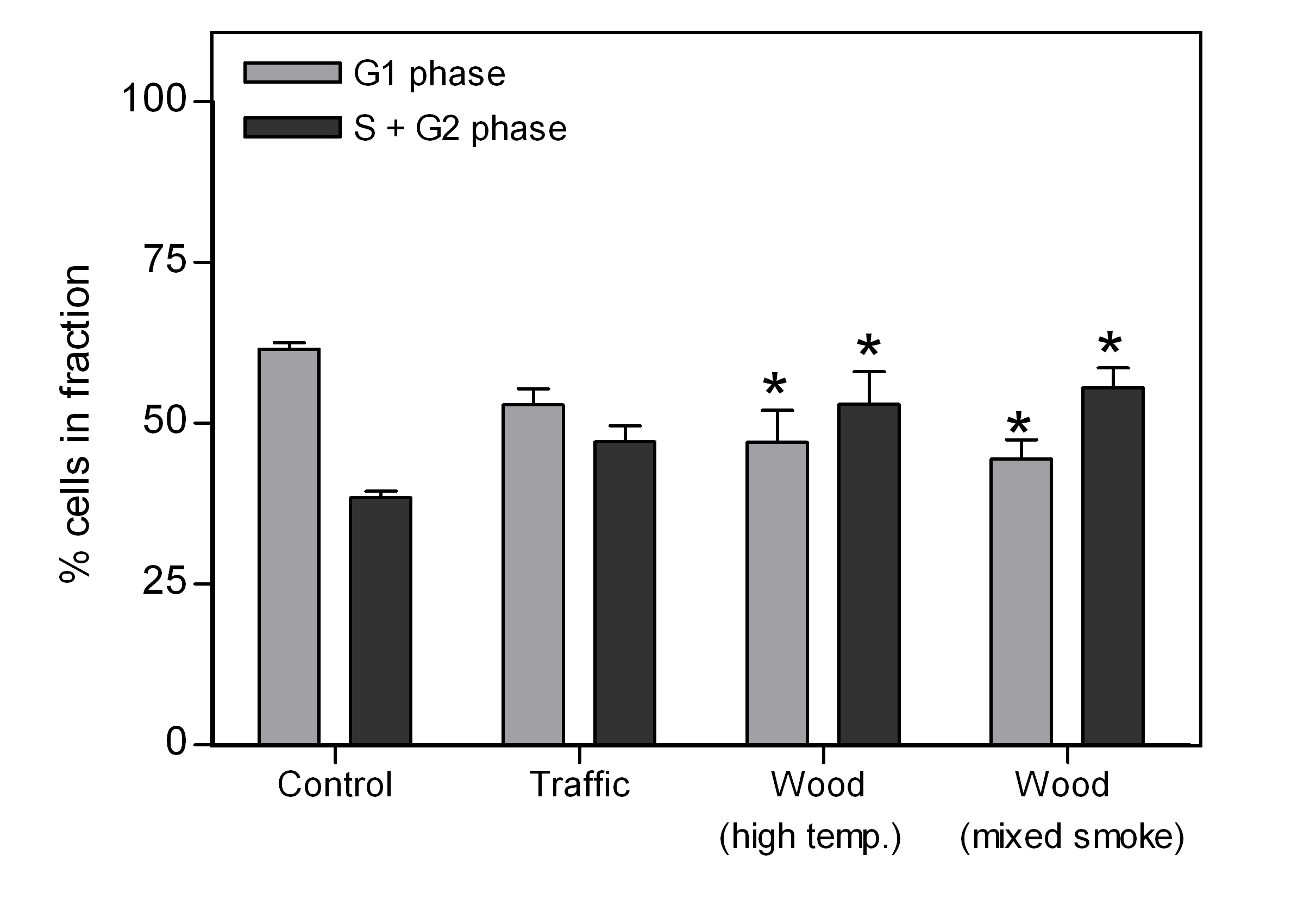
**

#### Figure 1: Cell cycle analysis. Co-cultures of pneumocytes (A549) and monocytes (THP-1) were exposed for 40 h to organic extracts at a concentration equivalent to 40 μg/cm^2^ of particles, before the flowcytometric cell cycle analysis was performed for THP-1 cells only. Histograms represent means ± SEM of separate experiments (n = 4).* p<0.05 exposed vs. control (two-way ANOVA with Bonferroni post-tests).

**Methodological description**

Particle- and extract-induced effects on cell cycle were analysed using a Becton and Dickenson (BD) LSRII flow cytometer with FacsDiva Software (version 6.1.2, BD Biosciences, San Jose, CA). After exposure to organic extracts the monocytes and pneumocytes were harvested for counting of cell numbers, but kept separately, and subsequently fixed in 0,2 % (w/v) paraformaldehyd (Aldrich/Sigma- Aldrich) before storage at + 4 °C until further processing. For cell cycle analysis the fixed cells were stained by Hoechst 33258 (B2883, Sigma-Aldrich) at a final concentration of 1 µg/ml. Monocytes and pneumocytes were analysed separately, since pilot studies showed that analysis of mixed samples caused two G1 peaks.

To obtain a measure of the proportion of cells in each phase of the cell cycle Multicycle AV DNA Analysis software (version 300, Phoenix Flow Systems, San Diego, CA) was used to do a curve fitting for the G1, S and G2 phases in the histograms achieved from the flow cytometry analysis.

**Particle- and extract-induced artefact in cell cycle analysis**

Pilot experiments revealed that wood smoke particles and the corresponding organic extracts caused a distortion of the cell cycle histogram obtained from A549 cells that could be due to an artefact introduced by organic compounds or the particles themselves (Figure 2). We observed an extreme broadening of the G1 peak to the left and right after exposure to wood

**Figure 2. Cell cycle analysis of A549 cells after exposure to native particles or organic extracts.** Cell cycle histograms obtained from A549 cells after 40 hours exposure to 40 ug/cm^2^ of wood smoke particles or equivalent concentrations of organic extract in co-culture with THP-1 cells.

smoke particles (Wood(high-temp)), but only to the right after exposure to the corresponding organic extract. Similar, though weaker tendencies of such effects were observed in THP-1 cells. Based on these data we chose to only carry out cell cycle analyses only for cells exposed to organic extracts in the remaining experiments.

To investigate whether the broadening of the G1 peak was an artefact or an actual effect on the cell cycle we also investigated the time-dependent effects on cell cycle patterns in monocultures of THP-1 cells exposed to one of the wood smoke extracts (Wood(high-temp)) (Figures 3 and 4). Figure 3 shows that the organic extract caused a broadening of the G1 peak already after 30 minutes exposure. Since effects on the cell cycle would not occur so rapidly, this indicates that the organic extract does indeed cause an artefact in the cell cycle analysis. In addition to a broadening of the G1 peak, the extract also lead to a shift of the G2 peak to the left providing a G2/G1 ratio of approximately 1.8, which is generally considered to be too

**Figure 3: Histograms showing time-dependent cell cycle effects of organic wood smoke extract on THP-1 cells.** 1.6 mill THP-1 monocytes were cultured in 2.4 ml medium and exposed to organic extract equivalent to 40 ug/cm^2^ for 0.5, 2 or 40 hours and harvested and analysed as described above.

low to be biologically relevant. The organic extract-introduced artefact could either be due to (i) organic compounds interfering with the binding of the Hoechst dye to the DNA or (ii) that the fluorescence activity of organic compounds in the samples (e.g. PAHs) interact with the fluorescence signal from the Hoechst dye.

Figure 4 shows results from the curve fitting with analytical settings chosen to adjust for the artefact. This was achieved by fixing the G2/G1 ratio to 1.8 for the histograms from cells exposed to organic extracts, and also performing the analysis for fixed CVs. After 30 min exposure, the proportions of cells in the G1 or S/G2 phases were similar in control and exposed cells. This suggests a successful adjustment of the curve fitting, since the data obtained from the curve fitting were not influenced by the distortion of the cell cycle histogram evident in Figure 3. The increase of cells in S/G2 phase observed after 2 and 40 hours is likely to be due to extract-induced effects on the cell cycle rather than the artefact, as these exposure times are high enough to expect cell cycle effects.


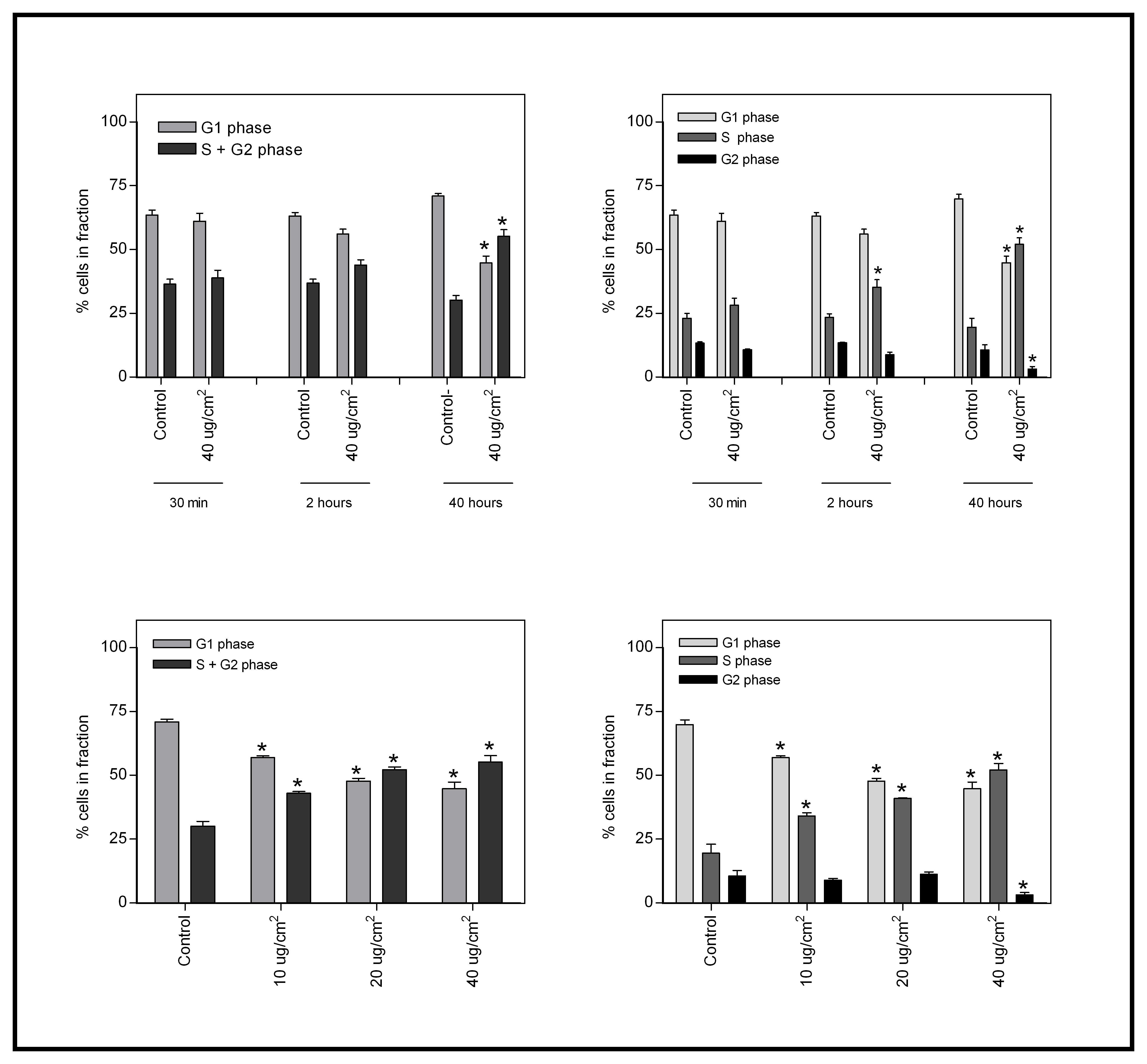


**Figure 4: Percent of THP-1 cells in different phases of cell cycle.** To account for the artefact introduced by the organic extract, the Multicycle analysis was performed with fixed G2/G1 ration and fixed CV for cells exposed to organic extract. For exposure conditions, see legend Figure 3 (in this file).

We did not have the opportunity to perform these time dependent experiments for the A549 pneumocytic cell line. The distortion of the histograms were more pronounced in the A549 cells than in the monocytic THP-1 cells, either because the extract had a more severe effect on the cells or due to more severe artefact effects. Since we were not confident that the analysis method applied above could account for artefacts introduced in the A549 cells, the histograms recorded from the exposed A549 cells were not analysed with Multicycle.
