# Supplementary material for: Wood smoke particles from different combustion phases induce similar pro-inflammatory effects in a co-culture of monocyte and pneumocyte cell lines
Source: Part Fibre Toxicol. 2012 Nov 23;9:45. doi: 10.1186/1743-8977-9-45 (PMC3544657; doi:10.1186/1743-8977-9-45)
Supplement: Additional file 5 — Cytokine binding to particles. [file 1743-8977-9-45-S5.doc]

**Additional file 5:**

**Cytokine binding to particles**

**Figure 1: Adsorption of IL-6 and IL-8 to a selection of particle samples.** Figures show the amount of cytokine remaining after 24 h incubation of 160µg/ml particles (equivalent to 40ug/ml) with a) 2000 or 4000 pg/ml IL-6 and b) 1000 or 3000 pg/ml IL-8. The experiments were performed according to the protocol described in [1]. Statistical analysis was performed by two-way ANOVA with Bonferroni post-test (n=3). No significant reduction in cytokine levels were observed after incubation with particles. This suggests that no cytokine binding occurs for these particle samples for the concentrations and exposure conditions relevant for the data presented in Figure 1-4 in the main paper.

**References**

1. Kocbach A, Totlandsdal AI, Låg M, Refsnes M, Schwarze PE: **Differential binding of cytokines to environmentally relevant particles: A possible source for misinterpretation of in vitro results?** *Toxicology Letters* 2008, **176:**131-137
