# Supplementary material for: Wood smoke particles from different combustion phases induce similar pro-inflammatory effects in a co-culture of monocyte and pneumocyte cell lines
Source: Part Fibre Toxicol. 2012 Nov 23;9:45. doi: 10.1186/1743-8977-9-45 (PMC3544657; doi:10.1186/1743-8977-9-45)
Supplement: Additional file 6 — Statistical comparison of cytokine release induced by the various particle samples. [file 1743-8977-9-45-S6.doc]

**Additional file 6:**

**Statistical comparison of cytokine release induced by the different particle samples**

H = the sample listed in the row is significantly higher than the sample listed in the column

L = the sample listed in the row is significantly lower than the sample listed in the column

**Release of TNF-α**

No significant differences between the samples

Release of IL-6

|  |  |  | |  | | **PM_0.1-2.5_** | | | | | | **PM_2.5-10_** | | | | | |
| --- | --- | --- | --- | --- | --- | --- | --- | --- | --- | --- | --- | --- | --- | --- | --- | --- | --- |
|  |  | Traffic | | Wood, high-temp. | | Wood, mixed- smoke | | Wood, start-up | | Wood, burn-out | | Wood, mixed- smoke | | Wood, start-up | | Wood, burn-out | |
|  |  |  |  |  |  |  |  |  |  |  |  |  |  |  |  |  |  |
|  |  | *12h* | *40h* | *12h* | *40h* | *12h* | *40h* | *12h* | *40h* | *12h* | *40h* | *12h* | *40h* | *12h* | *40h* | *12h* | *40h* |
|  | Traffic |  | | H | H | H |  | H |  | H |  |  |  | H | H | H |  |
|  | Wood, high-temp. |  | |  | |  | L |  | L |  | L |  | L |  | |  | |
| **PM_0.1-2.5_** | Wood, mixed- smoke |  | |  | |  | |  | |  |  |  | |  | |  | |
|  | Wood, start-up |  | |  | |  | |  | |  | | L |  |  | |  | |
|  | Wood, burn-out |  | |  | |  | |  | |  | |  | |  | H |  | |
| **PM_2.5-10_** | Wood, mixed- smoke |  | |  | |  | |  | |  |  |  | |  | |  | |
|  | Wood, start- up |  | |  | |  | |  | |  | |  | |  | |  | |
|  | Wood, burn- out |  | |  | |  | |  | |  | |  | |  | |  | |

Release of IL-8

|  |  |  | |  | | **PM_0.1-2.5_** | | | | | | **PM_2.5-10_** | | | | | |
| --- | --- | --- | --- | --- | --- | --- | --- | --- | --- | --- | --- | --- | --- | --- | --- | --- | --- |
|  |  | Traffic | | Wood, high-temp. | | Wood, mixed- smoke | | Wood, start-up | | Wood, burn-out | | Wood, mixed- smoke | | Wood, start-up | | Wood, burn-out | |
|  |  |  |  |  |  |  |  |  |  |  |  |  |  |  |  |  |  |
|  |  | *12h* | *40h* | *12h* | *40h* | *12h* | *40h* | *12h* | *40h* | *12h* | *40h* | *12h* | *40h* | *12h* | *40h* | *12h* | *40h* |
|  | Traffic |  | |  |  |  |  | H | H |  |  |  |  |  |  |  |  |
|  | Wood, high-temp. |  | |  | |  |  |  |  |  |  |  |  |  | |  | |
| **PM_0.1-2.5_** | Wood, mixed- smoke |  | |  | |  | | H | |  |  |  | |  | |  | |
|  | Wood, start-up |  | |  | |  | |  | |  | | L |  |  | |  | |
|  | Wood, burn-out |  | |  | |  | |  | |  | |  | |  |  |  | |
| **PM_2.5-10_** | Wood, mixed -smoke |  | |  | |  | |  | |  |  |  | |  | |  | |
|  | Wood, start-up |  | |  | |  | |  | |  | |  | |  | |  | |
|  | Wood, burn- out |  | |  | |  | |  | |  | |  | |  | |  | |
